# Etiology of community-acquired pneumonia in a population-based study: Link between etiology and patients characteristics, process-of-care, clinical evolution and outcomes

**DOI:** 10.1186/1471-2334-12-134

**Published:** 2012-06-12

**Authors:** Alberto Capelastegui, Pedro P España, Amaia Bilbao, Julio Gamazo, Federico Medel, Juan Salgado, Iñaki Gorostiaga, Maria Jose Lopez de Goicoechea, Inmaculada Gorordo, Cristobal Esteban, Lander Altube, Jose M Quintana

**Affiliations:** 1 Pneumology Service Hospital Galdakao, Galdakao, Bizkaia, Spain; 2 Basque Foundation for Health Innovation and Research (BIOEF) - CIBERESP, Sondika, Bizkaia, Spain; 3 Emergency Service Hospital Galdakao, Galdakao, Bizkaia, Spain; 4 Family Practice Comarca Interior Bizkaia, Galdakao, Bizkaia, Spain; 5 Microbiology Service Hospital Galdakao, Galdakao, Bizkaia, Spain; 6 Research Unit Hospital Galdakao -CIBERESP, Galdakao, Bizkaia, Spain; 7 Service of Pneumology, Hospital Galdakao, 48960, Galdakao, Bizkaia, Spain

**Keywords:** Pneumonia, Population-based study, Etiology

## Abstract

**Background:**

The etiologic profile of community-acquired pneumonia (CAP) for each age group could be similar among inpatients and outpatients. This fact brings up the link between etiology of CAP and its clinical evolution and outcome. Furthermore, the majority of pneumonia etiologic studies are based on hospitalized patients, whereas there have been no recent population-based studies encompassing both inpatients and outpatients.

**Methods:**

To evaluate the etiology of CAP, and the relationship among the different pathogens of CAP to patients characteristics, process-of-care, clinical evolution and outcomes, a prospective population-based study was conducted in Spain from April 1, 2006, to June 30, 2007. Patients (age >18) with CAP were identified through the family physicians and the hospital area.

**Results:**

A total of 700 patients with etiologic evaluation were included: 276 hospitalized and 424 ambulatory patients. We were able to define the aetiology of pneumonia in 55.7% (390/700). The most frequently isolated organism was *S. pneumoniae* (170/390, 43.6%), followed by *C. burnetti* (72/390, 18.5%), *M. pneumoniae* (62/390, 15.9%), virus as a group (56/390, 14.4%), Chlamydia species (39/390, 106%), and *L. pneumophila* (17/390, 4.4%). The atypical pathogens and the *S. pneumoniae* are present in pneumonias of a wide spectrum of severity and age. Patients infected by conventional bacteria were elderly, had a greater hospitalization rate, and higher mortality within 30 days.

**Conclusions:**

Our study provides information about the etiology of CAP in the general population. The microbiology of CAP remains stable: infections by conventional bacteria result in higher severity, and the *S. pneumoniae* remains the most important pathogen. However, atypical pathogens could also infect patients in a wide spectrum of severity and age.

## Background

Community-acquired pneumonia (CAP) is a common clinical disorder with an estimated incidence range from 1.6/1,000 to 16/1,000 per year [1-4]. The majority of pneumonia-related morbidity, mortality, and health care expenditures occur among persons who are hospitalized, however, 50 percent to 80 percent of adult patients with CAP are treated on an ambulatory basis [1,2,5,6].

Initial antibiotic selection for treatment of CAP is empirical, though a thorough understanding of the likely pathogens raises important questions regarding the current care of patients with CAP. Although the microbiology of CAP has probably remained relatively stable over the last decade, indeed could have changed over time due to the success of vaccinations and a population which is getting older, and sicker. Until now the majority of pneumonia etiologic studies are based on selected series of hospitalized patients, and there have been no recent, comprehensive population-based studies including hospitalized patients and outpatients treated by family physicians.

*Streptococcus pneumoniae* is the most common etiologic agent in hospitalized patients with CAP, particularly those with severe disease [7]. Some studies have found atypical bacteria to be the predominant microorganism for cases of ambulatory CAP [6] and for patients hospitalized with milder episodes of pneumonia [8]. However, population-based studies [1,9] have showed that *S. pneumoniae* was also the organism most frequently isolated in outpatients, and for each age group, the etiologic profile could be similar among inpatients and outpatients [9]. A better understanding of pathogens causer of CAP could lead to effective efforts to define their natural history and optimize the therapy.

The aim of the present study was to provide an assessment of the etiology of CAP that occurred in the adult population of a defined geographical area. Our second objective was to evaluate the relation that may exist between the different pathogens of CAP to patient characteristics, process-of-care, clinical evolution and outcomes.

## Methods

### Setting, design and study population

The Comarca Interior region, situated in the Basque Country (northern Spain), has a mixed urban, suburban, and rural population of 300,299 (254,523 aged ≥18). The age distribution, educational level, sources of employment, socioeconomic status, and health care services of the population are representative of the overall Basque Country [10]. Health care in this region is provided by the public network, which provides free unrestricted care to nearly 100% of the population.

We conducted a population-based study of all cases of adults (age ≥18 years) CAP within our area during a 15 months period. From April 1, 2006, to June 30, 2007, patients with CAP were recruited from a teaching hospital (Galdakao Hospital), the only hospital in the area, and 150 Family Practice (FP) physicians working in the Comarca Interior region. The adult cases of confirmed pneumonia that occurred during the study period were prospectively and consecutively enrolled in the study.

All eligible participating patients were informed of the study goals and gave informed consent to participate in the study. The project was approved by the ethics committee of Galdakao Hospital

### Definition of pneumonia

CAP was defined as pulmonary infiltrate on chest radiograph not known to be old and symptoms consistent with pneumonia, not acquired in a hospital or a nursing home residence. Patients were excluded if they were known to be positive for human immunodeficiency virus, were chronically immunosuppresed (defined as immunosuppression for solid organ transplantation, postsplenectomy, receiving ≥10 mg/day of prednisone or the equivalent for more than 30 days, treatment with other immunosuppressive agents, or neutropenia, i.e., <1.0 × 10^9^/L neutrophils), or who had been discharged from an acute care hospital or an on-site subacute care unit or from palliative care within the previous 14 days.

Cases were confirmed if there was a radiological finding suggestive of pneumonic infiltrate as reviewed by two members of the research team (PPE and AC).

### Measurements and management evaluation

Management for patients who initially visited an FP was left to the FP’s discretion. Patients attending the emergency department of Galdakao Hospital during the study period were managed according to a clinical guideline [11]. Clinical and demographic characteristics (age and sex) of each patient were recorded, along with all of the variables for the CURB-65 (**C**onfusion, **U**rea nitrogen, **R**espiratory rate, **B**lood pressure, age ≥**65** years) score [12] within the first 24 hours after diagnosis.

Process-of-care variables included the therapy employed (initial choice of antibiotic treatment, whether it was consistent with recommendations of the Spanish Society of Pneumology (SEPAR) [13] or the Infectious Diseases Society of America/American Thoracic Society (IDSA/ATS) guidelines [14], and antibiotics taken prior to admission), and the duration of antibiotic therapy.

Clinical evolution measures included treatment failure (defined as the development of clinical deterioration with hemodynamic instability, demonstrated respiratory failure or the appearance of it, required mechanical ventilation, demonstrated radiographic progression of pneumonia or the appearance of a new infectious foci, or had persistent fever or the reappearance of fever if change in treatment was needed) [15]; severe sepsis (defined as sepsis associated with organ dysfunction [16]); septic shock (defined as systolic arterial tension <90 mmHg and requirement of vasopressors for a minimum of 4 hours) [17]; hospitalization; admission to the intensive care unit (ICU); need for mechanical ventilation.

Outcome measures included vital status at 30 days after diagnosis; in-hospital mortality; hospital readmission within 30 days; length of hospital stay; length of time needed to return to normal daily activities; and subsequent hospitalization for patients initially treated as outpatients.

Vital status and readmission information for all patients were initially determined by telephone interviews up to 90 days after discharge. All reported deaths and dates of death were confirmed by a review of medical reports, public death registries, or both.

### Bacteriologic studies

The strategy for bacteriologic diagnosis included two blood cultures, a urinary antigen during the acute phase of the infection, and serological tests for atypical bacteria during the acute and remittance phases. A mixed infection was defined as pneumonia due to more than one pathogen.

In the first three days after diagnosis and 4 to 6 weeks thereafter, sera were collected, and tested for the presence of IgG and IgM to *Mycoplasma pneumoniae*, *Chlamydia pneumoniae*, *Chlamydophila psittaci, Coxiella burnetii,* and *Legionella pneumophila*. Antibodies to *M. pneumoniae, C. Burnetti,* and *L. pneumophila* were tested by immunofluorescent antibody assay and to *Chlamydophila spp* by microimmunofluorescence assay. Tests to detect influenza virus types A and B, parainfluenza virus types 1 to 3, adenovirus, and respiratory syncytial virus were performed with monoclonal antibodies for direct immunofluorescence assay. The collected urine samples were stored at –70°C or −20°C until testing. The detection of *L. pneumophila* was performed by means of an enzyme immunoassay system and *Streptococcus pneumoniae* by means of an immunochromatographic membrane assay (Binax Inc; Scarborough, ME) in a nonconcentrated urine sample.

An etiologic diagnosis was considered to be definitive if one of the following criteria were met: 1) isolation of respiratory pathogen in a sterile specimen (blood and pleural fluid); 2) four-fold rise in IgG titers for *M. pneumoniae*, *C. pneumoniae*, *C. psittaci*, *C. burnetii,* and *L. pneumophila*; 3) a single increased IgM titer for *M. pneumoniae* (≥1:16) or *C. pneumoniae* (≥1:10) or *C. psittaci* (≥1:32) or *C. burnetii* (≥1:64); 4) positive urinary antigen for *L. pneumophila* type 1 or *S. pneumoniae*; and 5) a positive result for one respiratory virus. An etiologic diagnosis was considered to be presumptive if a single increased IgG titer for *M. pneumoniae* (≥1:180), for *C. pneumoniae* (≥1:512), for *C. psittaci* (≥1:128), for *C. burnetii* (≥1:512), for *L.pneumophila* (≥1:256). In the present study the bacteriological examination of sputum was not compulsory, however, an etiologic diagnosis was considered to be presumptive if a validated sputum sample yielded one or more bacterial strains.

Since not all patients without etiologic diagnostic were evaluated by all diagnostic methods, the following minimal set of diagnostic tools was required for inclusion in the analysis: 1) urinary antigen and 2) paired serology or only 2° serology.

### Statistical analysis

Pathogens detected were compared among inpatients and outpatients, and according to age groups (18–44, 45–64, 65–74 and >74 years). Characteristics of patients, and process-of care and outcomes were compared in relation to etiologic categories. Chi-square and Fisher’s exact tests were performed for the comparison of categorical variables, and the analysis of variance or the nonparametric Kruskal-Wallis test were performed for continuous variables. The Cochran-Armitage test was performed for the trend analysis according to age groups.

Clinical evolution and outcomes were also compared according to etiologic categories adjusting by age, sex, and comorbidities. For continuous dependent variables (length of hospital stay and length of time needed to return to normal daily activities), the general linear model was used. Due to the non-normal distribution of these dependent variables, a log transformation was performed. Parameter estimates and standard errors were estimated after exponentiation. For all other dependent variables, all dichotomous, multivariate logistic regression models were performed. Odds ratios (OR) and 95% confidence intervals (95% CI) are presented.

All effects were considered significant at *P* < 0.05. All statistical analyses were performed using SAS for Windows statistical software, version 9.1 (SAS Institute, Inc., Carey).

## Results

A total of 700 patients with etiologic evaluation were included in our study: 276 hospitalized and 424 ambulatory patients. We were able to define the etiology of pneumonia in 390/700, 55.7% (310/390, 79.5% definitive diagnosis; 80/390, 20.5% presumptive diagnosis) (Table 1). The most frequently isolated organism was *S. pneumoniae* (170/390, 43.6%), followed by *C. burnetti* (72/390, 18.5%), *M. pneumoniae* (62/390, 15.9%), virus as a group (56/390, 14.4%), Chlamydia species (39/390, 106%), and *L. pneumophila* (17/390, 4.4%). Evidence of bacterial infection was present in 179 (179/360, 45.9%) episodes and atypical infection in 190 (190/390, 51.4%). Mixed infections were demonstrated in 9% (35/390). Results of sputum cultures were available for 12 cases. Pneumococcal infection was significantly more frequent among inpatients (64.8% vs. 22.2%; *P* < 0.0001). In contrast, atypical pathogens as a group were significantly more frequent among outpatients (67% vs. 30.6%; *P* < 0.0001). Among inpatients, the global incidence of *S. pneumoniae* was 64.8% (127/196) and that of atypical agent was 30.6% (60/196).

**Table 1:** Pathogens detected and methods used for diagnosis in 700 adults with community-acquired pneumonia

**Pathogens**			Diagnostic method performed^*^			
	**Inpatients (N = 276)**	**Outpatients (N = 424)**	**Urinary Antigen (N = 678)**	**Sputum (N = 222 )**	**Blood Culture (N = 221 )**	**Serologic Test (N = 663 )**
Any pathogen identified	196 (71)	194 (45.7)				
Conventional bacteria^†^	136 (69.4)	43 (22.2)				
*Streptococcus pneumoniae*^†^	127 (64.8)	43 (22.2)	155 (22.9)	8 (3.6)	24 (10.9)	
Others bacteria	9 (4.6)	0 (0)		4 (1.8)	5 (2.3)	
Atypical pathogen^†^	60 (30.6)	130 (67)				
*Coxiella burnetii*^†^	15 (7.7)	57 (29.4)				72 (10.9)
*Mycoplasma pneumoniae*	22 (11.2)	40 (20.6)				62 (9.4)
*Chlamydia pneumoniae*	11 (5.6)	26 (13.4)				37 (5.6)
*Chlamydia psittaci*	1 (0.5)	1 (0.5)				2 (0.3)
*Legionella pneumophila*^†^	11 (5.6)	6 (3.1)	17 (3.5)			
Virus	21 (10.7)	35 (18)				
*Influenza virus*	6 (3.1)	18 (9.3)				24 (3.6)
*Parainfluenza virus*	15 (7.7)	17 (8.8)				32 (4.8)
Total, mixed infection	21 (10.7)	14 (7.2)				

Data are presented as numbers (percentage) unless otherwise stated. Values for mixed infections are included in those for each of the infecting organism.

Others bacteria included *Escherichia coli* 4 cases, *Stafhylococcus aureus* 2 cases, *Enterococcus faecalis* 1 case, *Pseudomonas aeruginosa* 1 case, *Morganella morganii* 1 case*.*

^*^Data are presented as numbers of patients with positive results. % = positive results/number of test performed

^†^*P* value <0.05, between inpatients and outpatients.

Microbial etiology by age group is shown in Table 2. A trend toward a higher proportion at older ages was found for Pneumococcal infection (trend *P* < 0.0001). In contrast, for atypical infection a trend toward a low proportion at older ages was found (trend *P* < 0.0001). Virus infection as a group was significantly more frequent among patients aged ≥65 years than among patients aged <65 years (22.2% vs. 7%; *P* < 0.0001).

**Table 2:** Microbial etiology by age group in 700 adults with community-acquired pneumonia

**Pathogens**	**Age group**				
	**18–44 years (N = 205)**	**45–64 years (N = 181)**	**65–74 years (N = 120)**	**>74 years (N = 194)**	*P *value^*^
Any pathogen identified	121 (59)	80 (44.2)	67 (55.8)	122 (62.9)	0.0021
Conventional bacteria	28 (23.1)	31 (38.8)	33 (49.3)	87 (71.3)	<0.0001
*Streptococcus pneumoniae*	27 (22.3)	31 (38.8)	32 (47.8)	80 (65.6)	<0.0001
Others bacteria	1 (0.8)	0 (0)	1 (1.5)	7 (5.7)	0.03
Atypical agents	97 (80.2)	46 (56.3)	18 (26.9)	29 (23.8)	<0.0001
*Coxiella burnetii*	46 (38)	15 (18.8)	4 (6)	7 (5.7)	<0.0001
*Mycoplasma pneumoniae*	40 (33.1)	12 (15)	1 (1.5)	9 (7.4)	<0.0001
*Chlamydia pneumoniae*	8 (6.6)	8 (10)	10 (14.9)	11 (9)	0.3
*Chlamydia psittaci*	0 (0)	1 (1.3)	0 (0)	1 (0.8)	0.8
*Legionella pneumophila*	3 (2.5)	10 (12.5)	3 (4.5)	1 (0.8)	0.001
Virus	8 (6.6)	6 (7.5)	22 (32.8)	20 (16.4)	<0.0001
*Influenza virus*	5 (4.1)	4 (5)	7 (10.4)	8 (6.6)	0.4
*Parainfluenza virus*	3 (2.5)	2 (2.5)	15 (22.4)	12 (9.8)	<0.0001
Total, mixed infection	12 (9.9)	3 (3.8)	6 (9)	14 (11.5)	0.3

Data are presented as numbers (%) of patients, by age group. The rates of microorganism are related as the number of patients with any pathogen identified. Values for mixed infections are included in those for each of the infecting organism.

Others bacteria included *Escherichia coli* 4 cases, *Stafhylococcus aureus* 2 cases, *Enterococcus faecalis* 1 case, *Pseudomonas aeruginosa* 1 case, *Morganella morganii* 1 case*.*

^*^ The *p*-value obtained from the chi-square or Fisher exact test.

Characteristics of patients according to etiologic categories are provided in Table 3. In contrast with patients infected by atypical agents, those infected by conventional bacteria were elderly, had more comorbidities, or had a higher severity of CAP at diagnostic. The rate of patients immunized against pneumococcal infection was very low for all groups.

**Table 3:** Characteristics of patients with community-acquired pneumonia according to etiologic categories (N° = 390)

**Characteristics**	**Conventional Bacteria (N = 163 )**	**Atypical Agent (N = 151)**	**Virus (N = 41)**	**Mixed Infection (N = 35)**	***P *value**^**†**^
Age, years, mean (SD)	68.9 (18.3)	47.5 (18.3)	66.1 (17.5)	61.9 (22.9)	<0.0001
Female	72 (44.2)	51 (36.8)	19 (46.3)	9 (25.7)	0.07
Immunized against influenza	83 (53.6)	32 (22.2)	32 (82.1)	19 (54.3)	<0.0001
Immunized against pneumococcal infection	10 (6.8)	4 (2.8)	2 (5.1)	1 (2.9)	0.4
Number of comorbid conditions					0.002
0	96 (60)	120 (81.6)	24 (60)	21 (60)	
1	44 (27.5)	20 (13.6)	13 (32.5)	11 (31.4)	
>1	20 (12.5)	7 (4.8)	3 (7.5)	3 (8.6)	
Underlying diseases					
Neoplastic disease	10 (6.1)	4 (2.7)	0 (0)	3 (8.6)	0.2
Liver disease	1 (0.6)	2 (1.3)	0 (0)	3 (8.6)	0.005
Congestive heart failure	8 (4.9)	4 (2.7)	0 (0)	0 (0)	0.2
Cerebrovascular disease	11 (6.8)	2 (1.3)	1 (2.4)	2 (5.7)	0.09
Renal disease	7 (4.3)	4 (2.7)	1 (2.4)	2 (5.7)	0.7
Chronic obstructive pulmonary disease	34 (21.1)	9 (6.1)	11 (27.5)	4 (11.4)	0.0003
Diabetes mellitus	18 (11.3)	13 (8.8)	6 (14.6)	3 (8.6)	0.7
Severity of illness at diagnostic					
CURB-65 score^*^					<0.0001
0-1	79 (48.5)	131 (86.8)	29 (70.7)	20 (57.1)	
2	47 (28.8)	13 (8.6)	9 (22)	10 (28.6)	
>2	37 (22.7)	7 (4.6)	3 (7.3)	5 (14.3)	
Bilateral or multilobe radiographic involvement	38 (23.5)	7 (4.7)	0 (0)	2 (5.7)	<0.0001

SD = standard deviation.

Data are presented as numbers (percentage) of patients unless otherwise stated. Percentages exclude patients with missing data.

^*^ Severity of illness on admission assessed with CURB-65 score (Confusion, Urea nitrogen, Respiratory rate, Blood pressure, age ≥65 year).

^†^ The *p*-value obtained from the Chi-square or Fisher exact test for categorical variables, and Kruskal-Wallis test for continuous variables.

Process-of-care is shown in Table 4. First-line antibiotic therapy was a monotherapy in 92.8% patients. Antibiotics initially prescribed were overcoat quinolones (60%) for all etiologic categories. Almost a quarter of CAP by atypical agents were treated with betalactam antibiotics.

**Table 4:** Process-of-care of patients with community-acquired pneumonia according to etiologic categories (N° = 390)

**Process-of-care**	**Conventional Bacteria (N = 163)**	**Atypical Agents (N = 151)**	**Virus (N = 41)**	**Mixed Infection (N = 35)**	***P *value**^**║**^
Antibiotics prescribed					0.0004
Betalactam	39 (23.9)	33 (22.3)	9 (22.5)	4 (11.4)	
Macrolide	3 (1.8)	24 (16.2)	5 (12.5)	3 (8.6)	
Betalactam/macrolide	19 (11.7)	5 (3.4)	4 (10)	0 (0)	
Fluoroquinolone	99 (60.7)	85 (57.4)	22 (55)	28 (80)	
Others	3 (1.8)	1 (0.7)	0 (0)	0 (0)	
Taking antibiotics prior to diagnosis	25 (16.1)	27 (18.9)	7 (18)	7 (20.6)	0.9
Appropriate antibiotic therapy according to Spanish guidelines^*^	121 (74.2)	89 (60.1)	26 (65)	28 (80)	0.02
Appropriate antibiotic therapy according to American guidelines^*^	123 (75.5)	113 (76.4)	31 (77.5)	31 (88.6)	0.4
Antibiotics within 8 hours^†^	111 (91.7)	34 (87.2)	8 (61.5)	21 (100)	0.002
Duration of antibiotic therapy after diagnosis, mean (SD), days^‡^	10 (2.5)	10.3 (2.1)	10.2 (1.5)	9 (2.6)	0.03
Duration of intravenous therapy after diagnosis, mean (SD), days^†‡^	3.2 (3)	2.4 (2.1)	2.5 (1.8)	2.2 (1.4)	0.3

SD = standard deviation.

Data are presented as numbers (percentage) of patients unless otherwise stated. Percentages exclude patients with missing data. Treatment failure, septic shock, and severe sepsis are defined in text.

^*^Appropriate antibiotic according to Spanish guidelines (reference 13). Appropriate antibiotic according American guidelines (reference 14).

^†^ Only for hospitalized patients.

^‡^ Inhospital deaths are excluded.

^║^ The *p*-value obtained from the Chi-square or Fisher exact test for categorical variables, and Kruskal-Wallis test for continuous variables.

Clinical evolution and outcomes are provided in Table 5. In contrast with patients infected by atypical agents, those infected by conventional bacteria had a higher hospitalization rate, showed worse evolution and higher length of hospital stay . However, among patients infected by atypical agents 25.8% needed hospitalization with an inhospital mortality rate of 5.1%. There was no difference according to etiologic categories for inhospital mortality, readmission within 30 days, and the mean duration of return to daily activity. No deaths were found for patients with mixed, virus or *L. pneumophila* infections, and none of them were admitted to an ICU. After adjusting by sex, age, and comorbidities, the previous analyses yielded similar results (Table 6).

**Table 5:** Clinical evolution and outcomes of patients with community-acquired pneumonia according to etiologic categories (N° = 390)

**Clinical evolution and outcomes**	**Conventional Bacteria (N = 163)**	**Atypical Agents (N = 151)**	**Virus (N = 41)**	**Mixed Infection (N = 35)**	***P *value**^║^
**Clinical evolution**					
Treatment failure	15 (9.2)	8 (5.3)	0 (0)	1 (2.9)	0.1
Severe sepsis	71 (43.8)	22 (14.6)	6 (14.6)	6 (17.1)	<0.0001
Septic shock	8 (4.9)	1 (0.7)	0 (0)	0 (0)	0.04
Hospitalized	123 (75.5)	39 (25.8)	13 (31.7)	21 (60)	<0.0001
Admission to intensive care unit	14 (8.6)	1 (0.7)	0 (0)	0 (0)	0.0007
Use of mechanical ventilation	2 (1.2)	1 (0.7)	0 (0)	0 (0)	0.8
**Outcomes**					
Mortality within 30 days	7 (4.3)	2 (1.3)	0 (0)	0 (0)	0.2
In-hospital mortality^*^	5 (4.1)	2 (5.1)	0 (0)	0 (0)	0.7
Readmission within 30 days^*^	10 (8.1)	2 (5.1)	0 (0)	2 (9.5)	0.7
Length of hospital stay^*†^ (days)					
Mean (SD)	3.9 (3.1)	3 (2.7)	2.9 (2)	2.9 (2.4)	0.05
>3	57 (48.3)	8 (21.6)	4 (30.8)	5 (23.8)	0.01
Return to daily activity^†^, mean (SD),days	19.5 (9.1)	19.6 (11)	21.6 (11.5)	17.8 (11.1)	0.3
Subsequent hospitalization^‡^	1 (2.6)	3 (2.7)	1 (3.6)	1 (7.1)	0.8

SD = standard deviation.

Data are presented as numbers (percentage) of patients unless otherwise stated. Percentages exclude patients with missing data. Treatment failure, septic shock, and severe sepsis are defined in text.

^*^ Only for hospitalized patients.

^†^Inhospital deaths are excluded.

^‡^ Only for ambulatory patients.

^║^ The *p*-value obtained from the Chi-square or Fisher exact test for categorical variables, and Kruskal-Wallis test for continuous variables.

**Table 6:** Adjusted comparisons of clinical evolution and outcomes of patients with community-acquired pneumonia according to etiologic categories (N° = 390)

**Clinical evolution and outcomes**	**Conventional Bacteria vs. Others**^**††**^		**Conventional Bacteria vs. Atypical Agents**^**††**^	
	**OR (95% CI)**	***P *value**	**O7R (95% CI)**	***P *value**
**Clinical evolution**				
Treatment failure	2.3 (0.9–5.8)	0.1	1.5 (0.6–4.2)	0.4
Severe sepsis	3.0 (1.8–5.1)	<0.0001	2.3 (1.2–4.4)	0.01
Septic shock	15.9 (1.8–142.5)	0.01	11.8 (1.3–109.6)	0.03
Hospitalized	4.5 (2.7–7.5)	<0.0001	5.1 (2.8–9.2)	<0.0001
Admission to intensive care unit	27.9 (3.4–227.9)	0.002	19.5 (2.3–164.1)	0.006
Use of mechanical ventilation	6.0 (0.5–76.8)	0.2	4.6 (0.3–61.7)	0.2
**Outcomes**				
Mortality within 30 days	2.2 (0.4–11.6)	0.4	0.8 (0.1–4.7)	0.8
In-hospital mortality^*^	1.1 (0.2–6.3)	0.9	0.3 (0.04–2.4)	0.3
Readmission within 30 days^*^	1.4 (0.4–4.9)	0.6	1.7 (0.3–9.2)	0.5
Length of hospital stay^*†^ (days)				
Mean (SD)	1.2 (1–1.6)^**║**^	0.05	1.2 (0.9–1.7)^**║**^	0.2
>3	2.7 (1.3–5.3)	0.005	3.2 (1.2–8.2)	0.02
Return to daily activity^†^, mean (SD),days	1.0 (0.9–1.2)^**║**^	0.6	1.1 (0.9–1.3)^**║**^	0.5
Subsequent hospitalization^‡^	0.7 (0.1–6.5)	0.8	0.8 (0.1–8.7)	0.9

OR = Odds ratio; CI = Confidence interval.

Treatment failure, septic shock, and severe sepsis are defined in text.

^*^ Only for hospitalized patients.

^†^Inhospital deaths are excluded.

^‡^ Only for ambulatory patients.

^**††**^Adjusted analyses were performed comparing the clinical evolution and outcomes between Conventional Bacteria vs. the rest of groups, and between Conventional Bacteria vs. Atypical Agents, adjusting for age, sex and comorbidities.

^║^For these two continuous dependent variables, the general linear model was used, and due to the non-normal distribution of these variables, a log transformation was performed. Parameter and standard errors were estimated after exponentiation. Therefore, the parameter estimated is the ratio between the length of hospital stay or the length to return to daily activities of Conventional Bacteria vs. the reference group.

All the patients who passed away, both in hospital as well as at 30-days had a definitive etiologic diagnostic. Out of all patients admitted at the ICU [15], for 14 (Table 5) the diagnostic was definitive, while 1 patient was not diagnosed etiologically: blood culture, negative; urinary antigen, negative; sputum culture, negative; serologic test 1° and 2°, negative. Out of all patients with septic shock [10], for 9 (Table 5) the diagnostic was definitive, while 1 patient was not diagnosed etiologically: blood culture, negative; urinary antigen, negative; sputum culture, negative; serologic test 1° and 2°, negative. Out of all patients with severe sepsis (131), for 105 (Table 5) the diagnostic was definitive, while 26 patients were not diagnosed etiologically: blood culture, negative for 17 (9 were not done); urinary antigen, negative for all; sputum culture, negative for 5 (21 not done); serologic test 2nd sample, negative for all, serologic test 1st sample, negative in 16 (10 not done).

## Discussion

In our population-based study we assessed the etiology for CAP, in search of evidence and insight into the different roles and pathlines concerning diverse pathogens. Our results confirm many already well documented features, i.e. – *S. pneumoniae* was the most common pathogen, causing a greater degree of severity. We bring to light as a new finding the fact that, viruses, mixed and *L. pneumophila* infections do not imply greater severity. What is more, atypical pathogens and the *S. pneumoniae* are found in a wide spectrum of severity and age levels. Our findings emphasize the part that atypical organisms play.

The study contains distinctive high points. It is a prospective population-based survey that include***s*** all cases of CAP occurrence in the overall total population of a defined geographical area, and consequently avoids any selection bias. We surveyed a large number of hospitalized and ambulatory unselected adult patients for whom we had gathered highly detailed data. Furthermore, our study is original in that it links the etiology to the clinical evolution and the consequent outcomes.

In agreement with other studies *S. pneumoniae* is still the predominant infection [1-3,18,19]. It was the most common agent in hospitalized patients and the second most common cause of illness in outpatients. *C. burnetti* had unusual frequencies in our study due to regional epidemiological factors which are markedly different from those in other areas of the western world [20,21]. In our region the sheep are the usual reservoir and the infection source for other domestic and wildlife species and the human population [22]. *M. pneumoniae* was the third most common pathogen isolated, although it was less common than in some earlier studies [23,24] probably due to cyclicity of *Mycoplasma* epidemics. Consistent with previous studies [1,19,25], the frequency of *L. pneumophila* infection was very low (<2%) and was not implicated in greater severity. The mixed pneumonia group is a heterogeneous group in which proportions varied among studies [9,26]. We found mixed infections in 9% of patients, and, like others [19], nearly all of our cases were non-severe CAP, contrary to the view that mixed infections can be severe [27,28]. Viral infections were attributable to old age patients with chronic obstructive pulmonary disease. In contrast with previous studies [29], viral etiology was associated with low severity of illness, possibly related to the high rate of influenza immunization in this group. In previous population-based studies about CAP in adult population [1-3]*H. influenzae* infection has been diagnosed in 1%-10%. The widely varying results are more likely to reflect differences in the bacteriologic methods than true differences in the etiology. In the present study the bacteriological examination of sputum was not compulsory. This approach implicated a failure to detect the true incidence of *H. influenza, S. aureus*, and *P. aeruginosa*. Moreover, the infection by *S. aureus* is always very infrequent in CAP, especially among outpatients.

According to etiologic categories the profile of patients is similar to the combined information from previous studies of CAP [1-4,8]: infections by atypical agents were characterized by milder episodes, while more severe infections were caused by conventional bacteria. In fact, patients with conventional bacteria have higher hospitalization rate, higher CURB65 scores at admission, more bilateral radiographic involvement and more adverse clinical evolution and outcomes such as severe sepsis, septic shock, ICU admission and longer length of stay. However, our findings confirm that atypical pathogens and the *S. pneumoniae* are present in pneumonias of a wide spectrum of severity and age, as had been stated in a previous study [30,31]. Almost 26% of patients infected with any atypical agents needed to be hospitalized and, like others [32], atypical pathogens were present in a considerable proportion of inpatients with CAP (30.6%). Conversely, among patients with conventional bacteria almost 25% were treated at home with excellent evolution. In the present study the mortality can be compared between conventional bacteria and those atypicals who require a convalescent serum, because all the patients who passed away, both in hospital as well as at 30-days, has a definitive etiologic diagnostic. Therefore, the assessment of this outcome is not biased towards a better outcome for atypical organisms.

In our study antibiotic treatment was consistent with the Spanish guideline strategy [13], which recommends targeting pneumococcal and atypical infection for inpatients and outpatients. Likewise, the IDSA/ATS [14] guideline strategy recommends empirical antibiotic treatment to cover typical and atypical pathogens. Our data show that *S. pneumoniae* as well as atypical agents were causing pneumonia in hospitalized and ambulatory patients of all ages, questioning the empirical use of an antibiotic to cover atypical and not atypical agents in ambulatory pneumonia. There is concern about this question because evidence is lacking that clinical outcomes are improved by using antibiotics against atypical pathogens in all-cause non-severe CAP [33], and a greater dependence on quinolones as initial therapy raises concerns regarding the potential for the development of antibiotic resistance.

Our study has some limitations. We made a great effort to identify all patients with CAP in the catchment area: all local health care centers and the referral hospital participated in the reporting of patients; all primary health care centers were closely supervised by a local FP, and all information was coordinated by a member of the research team. However, full participation of all 150 FP was not obtained and, therefore, we must assume as a limitation the fact that not all real cases have been included. As in most pneumonia studies [1,6,25,29,34], specific etiologic diagnoses could not be assigned for 44.3% of the patients enrolled in the study. It is difficult to collect quality sputum samples from outpatients. Likewise, it is difficult to obtain blood cultures from outpatients. The absence of sputum cultures in the strategy of bacteriologic diagnosis and the lack of molecular testing for both conventional and atypical organisms are other limitations of the study.

## Conclusions

Our study updates the information concerning the epidemiology and management of CAP. *S. pneumoniae* remains the most important pathogen, and almost 50% of the patients were infected with *C. burnetti, M. pneumoniae,* Chlamydia sp, or *L. pneumophila*. Although all identified agents infect patients of a wide spectrum of severity and age, infection by conventional bacteria showed higher severity, and there is a high incidence of *S. pneumoniae* in elderly patients.

## Competing interest

The authors declare that they have no competing interests.

## Authors’ contributions

AC, PPE, JJG, FM, JS, IG and JMQ conceived and designed the study. JJG, FM, JS, IG, IM, CE and LA enrolled patients and collected and compiled data. MJLG performed the bacteriologic studies. AB performed the statistical analysis. AC, PPE, AB, MJLG and JMQ analyzed and interpreted the data. AC, PPE and JMQ wrote the report. AB, JJG, FM, JS, IG, MJLG, IM, CE and LA commented and revised the report. All authors read and approve the final manuscript.

## Pre-publication history

The pre-publication history for this paper can be accessed here:

http://www.biomedcentral.com/1471-2334/12/134/prepub
